# A Multi-Evidence Approach to the Systematics of the Genus *Satyrium* Sw. Based on Time-Calibrated Phylogeny, Morphology, and Biogeography

**DOI:** 10.3390/ijms27010453

**Published:** 2025-12-31

**Authors:** Natalia Olędrzyńska, Sławomir Nowak, Aleksandra M. Naczk, Marcin Górniak, Dariusz L. Szlachetko

**Affiliations:** 1 Department of Evolutionary Genetics and Biosystematics, Faculty of Biology, The University of Gdansk, Wita Stwosza 59, 80-308 Gdansk, Poland; 2 Department of Plant Taxonomy and Nature Conservation, Faculty of Biology, The University of Gdansk, Wita Stwosza 59, 80-308 Gdansk, Poland

**Keywords:** biogeography, classification, hybrid species, orchids, plant taxonomy

## Abstract

The genus *Satyrium* (Orchidaceae) is a large, mostly sub-Saharan genus with a single species reported from Madagascar and Asia. Taxonomical complexity and high morphological diversity make the classification within the genus difficult to handle. In this study, we attempted to solve this problem using a comprehensive approach based on data from multiple sources. We combined morphological data from vegetative parts with data on flower structure using timescale phylogenetics conducted for both nuclear internal transcribed spacer (ITS) and plastid markers (*matK*, *trnS-trnG*, *trnL*, *trnL-trnF*). Phylogenetic studies confirmed most of the results of previous studies and led to the identification of six potential hybridization events within the genus. Morphological diversity often does not correspond to phylogenetic relationships within the genus, and many evolutionary lineages began to diverge only at the end of the early Miocene and in the late Miocene. The development of similar characteristics is the result of this diversification under the influence of similar environmental pressures. Reconstruction of the historical geographical range of *Satyrium* showed that the regions of South Africa and the mountainous areas of Eastern Africa played the most important role in the diversification of the genus.

## 1. Introduction

The terrestrial orchid genus *Satyrium* Sw., comprising about 90 species [1,2], is widely distributed in temperate and montane regions of Sub-Saharan Africa, with the greatest diversity observed in southern Africa, especially in the Cape Floristic Region, which is a well-known biodiversity hotspot [3,4]. Several species have also been recorded in Madagascar and Asia, e.g., [5].

**Figure 1:**
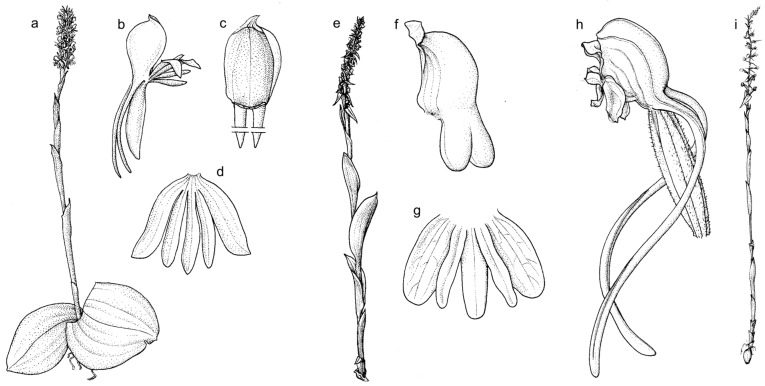
The morphologically diverse *Satyrium* species: *S. orbiculare* Rolfe with leaves adpressed to the ground (**a**), filiform spur (**b**), globose lip (**c**) and other flower segments (**d**); *S. amblyosaccus* Schltr. with opposite leaves (**e**), cochleate lip and saccate spur (**f**), and other flower segments (**g**); *S. chlorocorys* Rolfe with spur longer than ovary (**h**) and leaves gathered in the lower part of the stem (**i**). Drawn from *Lisowski* et al. *10569* (**a**–**d**; UGDA), *Stolz 2551* (**e**–**g**; K), and *Young 1344* (**h** and **i**; BM) [24].

The classification of *Satyrium* at both higher and infrageneric levels has changed over the years. It has been placed within different subtribes and tribes, e.g., [6,7,8,9,10,11], although these classifications were not supported by molecular data [5,12,13,14]. More recent phylogenetic studies [1,13,15,16,17,18] indicate that this genus is related to the subtribe Orchidinae s.l., where it forms a separate basal lineage. The enormous morphological diversity of *Satyrium* species makes its infrageneric classification extremely difficult (Figure 1). Various authors, relying solely on morphological data, have attempted to divide the genus into subgenera or sections [19,20,21,22,23].

In 2005, van der Niet et al. [1] presented the first molecular framework for infrageneric classification, identifying five informal clades within the genus *Satyrium* (*Satyrium*, *Chlorocorys*, *Macrophylla*, *Trinervia* and *Brachysaccium*). The strong cytonuclear discordance observed in Asian representatives of the genus (*S. nepalense, S. yunnanense*) prevented its confident placement, as these species are grouped differently in nuclear and plastid analyses. Moreover, a further study [25] revealed extensive topological incongruence throughout the genus and suggested a potential hybrid origin for several species, although limited nuclear sampling prevented a definitive conclusion from being drawn. To date, the ITS remains the only nuclear marker studied. Therefore, making a final decision on the infrageneric classification of this group based on molecular data, which is additionally characterised by cytonuclear discordance, remains a serious challenge. Our aim was to improve the understanding of the classification of *Satyrium* by integrating molecular, morphological, and biogeographic data. In this paper, we discuss the general morphology of *Satyrium* in relation to phylogenetic studies to understand the discrepancies between the results and the possibility of incorporating them into a classification system. We rely on the estimated time of diversification within the genus and its biogeography. Therefore, we present the timescale for *Satyrium* based on nuclear and combined plastid data. We also highlight the origin and affinity of Asian representatives of the genus, and its previously recognized clades are discussed, considering their morphological characteristics. In addition, we reconstruct the biogeographical history of the genus and review the possible range of ancestors, expansion patterns, and current distribution.

## 2. Results

### 2.1. Phylogenetic Analyses

**Figure 2:**
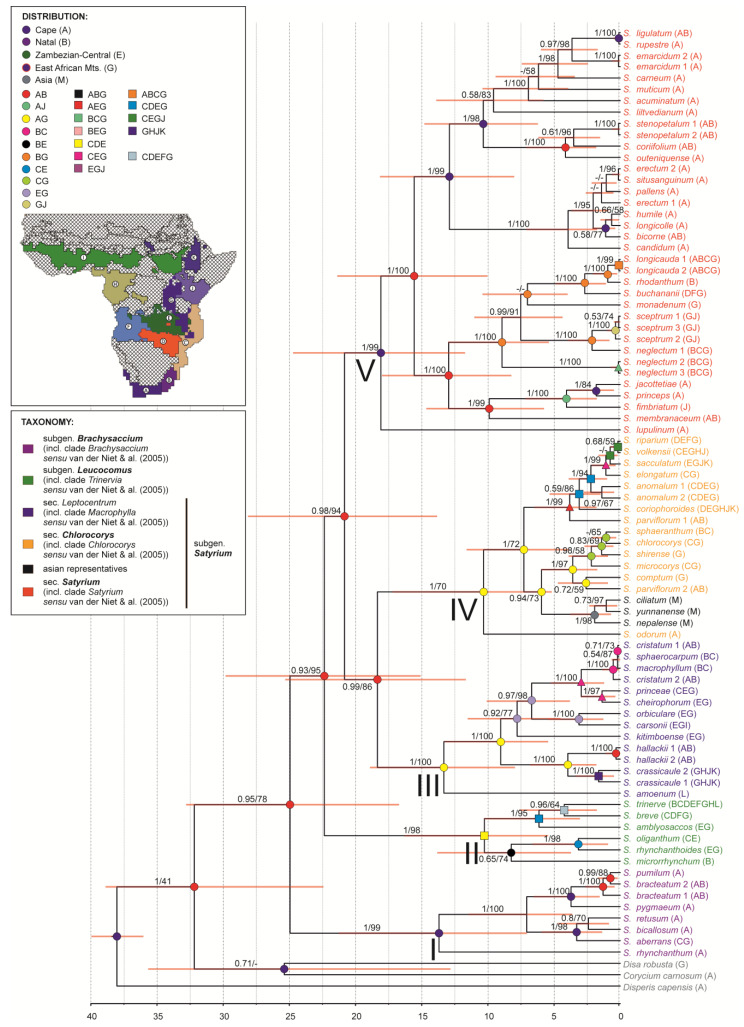
The maximum clade credibility tree for *Satyrium*, based on the ITS nuclear marker, obtained using Bayesian inference. Numbers above branches indicate posterior probability and bootstrap support values from maximum likelihood analysis (PP/BS). The divergence time is given in millions of years ago (Mya) at the bottom. Horizontal bars at nodes, shown in orange, represent 95% highest posterior density (HPD) intervals of divergence times. Taxonomic treatment as well as ancestral geographical ranges obtained from a BayArea model are given in different colours, according to the legend.

**Figure 3:**
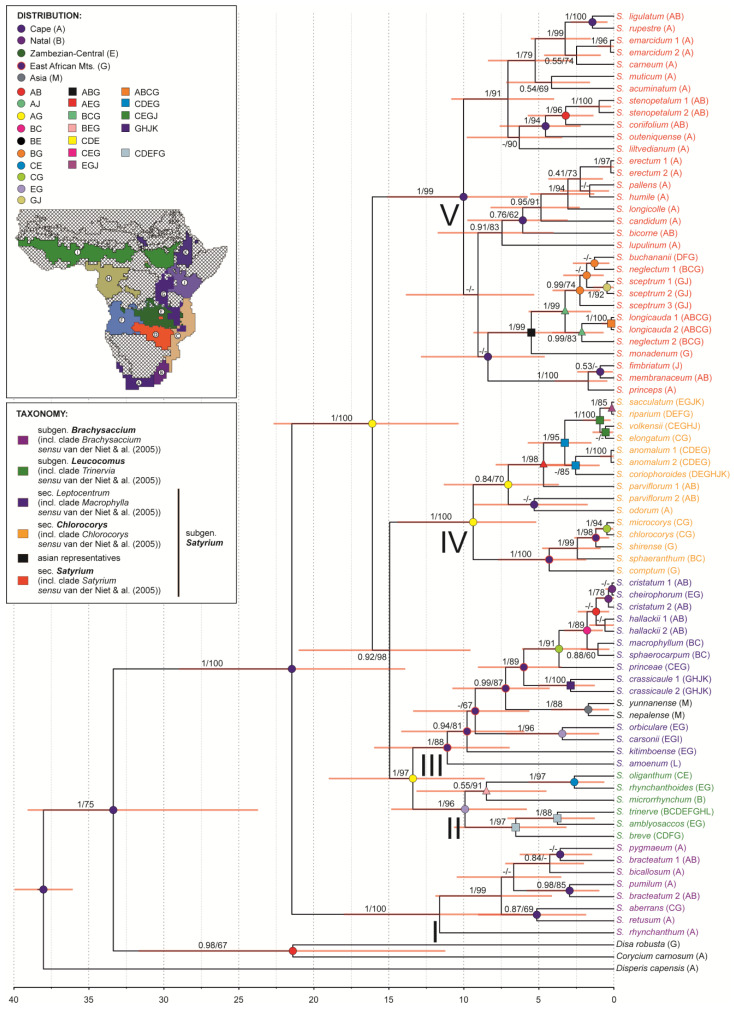
The maximum clade credibility tree for *Satyrium*, based on a combined plastid dataset (*matK*, *trnS*-*trnG* intergeneric spacer, *trnL*-*trnF* intergeneric spacer and *trnL* intron), obtained using Bayesian inference. Numbers above branches indicate posterior probability and bootstrap support values from maximum likelihood analysis (PP/BS). The divergence time is given in millions of years ago (Mya) at the bottom. Horizontal bars at nodes, shown in orange, represent 95% highest posterior density (HPD) intervals of divergence times. Taxonomic treatment as well as ancestral geographical ranges obtained from a BayArea model are given in different colours, according to the legend.

We used trees from molecular clock analyses to present our results. Although more species were analysed in this study, ITS and plastid analyses yielded essentially similar topologies (Figure 2 and Figure 3), consistent with previous studies [1,25]. Five major clades were recovered within the genus *Satyrium.* However, several nodes showed a significant cytonuclear incongruence.

Clade I (corresponding to *Brachysaccium sensu* van der Niet et al. [1]) was monophyletic and consistently recovered as the basal lineage in both datasets. The most recent common ancestor (MRCA) of this group occurred 13.69–11.62 Mya (PP = 1/BS = 99–100).

Clade II (corresponding to *Trinervia sensu* van der Niet et al. [1]) was also monophyletic (tMRCA: 10.26–9.92 Mya; PP = 1/BS = 96–98) in both analyses, but its placement differed as ITS data recovered it as an early diverging lineage (tMRCA: 22.36 Mya; PP = 0.93/BS = 95), while plastid data placed it at the one node with clade III and Asian species (tMRCA: 13.4 Mya; PP = 1/BS = 97).

Clade III (corresponding to *Macrophylla sensu* van der Niet et al. [1]) and clade IV (corresponding to *Chlorocorys sensu* van der Niet et al. [1]) showed the strongest topological conflict. ITS analyses supported the monophyly of clade III (tMRCA: 13.33 Mya; PP = 1/BS = 100), while plastid data supported only clade IV (tMRCA: 9.38 Mya; PP = 1/BS = 100). The Asian species (*S. nepalense* and *S. yunnanense*) were placed in clade IV in the ITS tree but grouped within clade III in the plastid tree. Clade V (corresponding to *Satyrium* clade *sensu* van der Niet et al. [1]) was well supported in both trees (tMRCA: 18.08–10.02 Mya; PP = 1/BS = 99), although its sister group relationships differed between analyses.

Overall, several nodes remained unresolved within the genus, and conflicts between ITS and plastid topologies were common, particularly among clades II-IV. Only clade I showed complete concordance between the analysed datasets. These patterns are likely associated with low phylogenetic signal and the limited nuclear dataset (only ITS were analysed), which limits resolution at deeper and intermediate nodes.

### 2.2. Plastid–Nuclear Discordance

**Figure 4:**
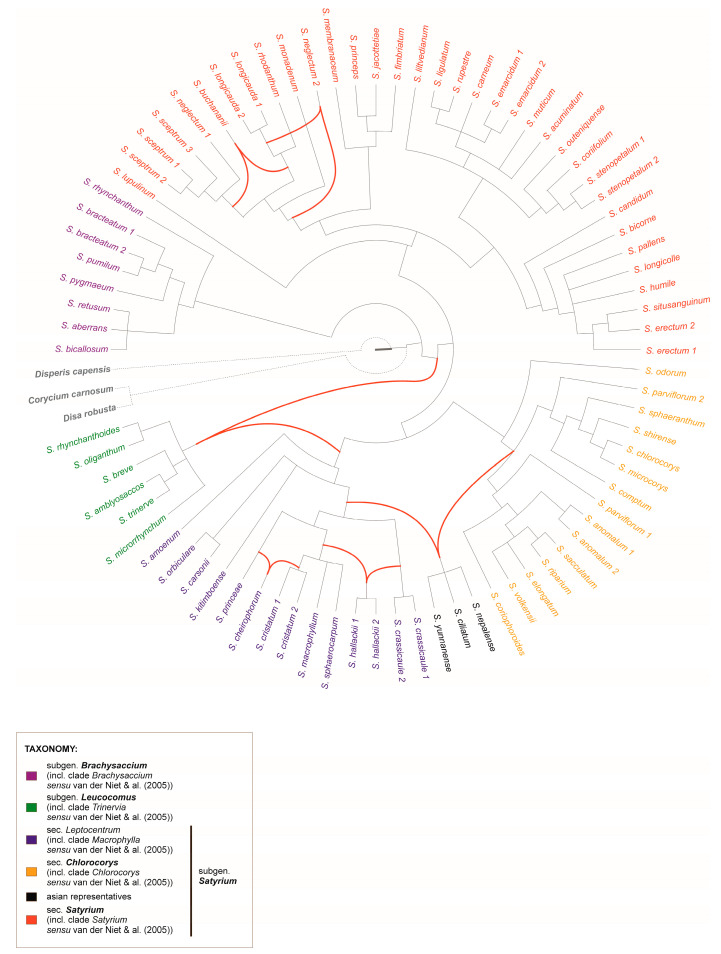
The hybridization network based on two incongruent Bayesian trees (ITS and plastid combined), where nodes with a PP value lower than 0.98 were removed. Red lines point to conflicts between both trees.

Based on the analysis of the topology of the phylogenetic trees obtained in this study (Figure 1 and Figure 2), after removing nodes with support below 0.98 PP, a network was constructed (Figure 4) that reveals several topological conflicts among lineages of *Satyrium*. These conflicts are observed both between plastid and nuclear datasets and within particular clades, suggesting instances of phylogenetic incongruence. It is worth noting that discrepancies occur in the placement of *S. hallackii*, *S. cheirophorum*, *S. buchananii*, and one individual of *S. neglectum*, as well as within the group of Asian species (*S. nepalense*, *S. ciliatum*, and *S. yunnanense*) and clade III (corresponding to *Macrophylla* clade *sensu* van der Niet [1]). These patterns indicate possible reticulation or incomplete lineage sorting, which is discussed in more detail in the Section 3.

### 2.3. Morphological Similarity

To describe the morphological relationships between *Satyrium* species, the resulting similarity matrix was used in the cluster analysis. The morphological clustering cophenetic coefficient with Jaccard’s similarity matrix was 0.809.

**Figure 5:**
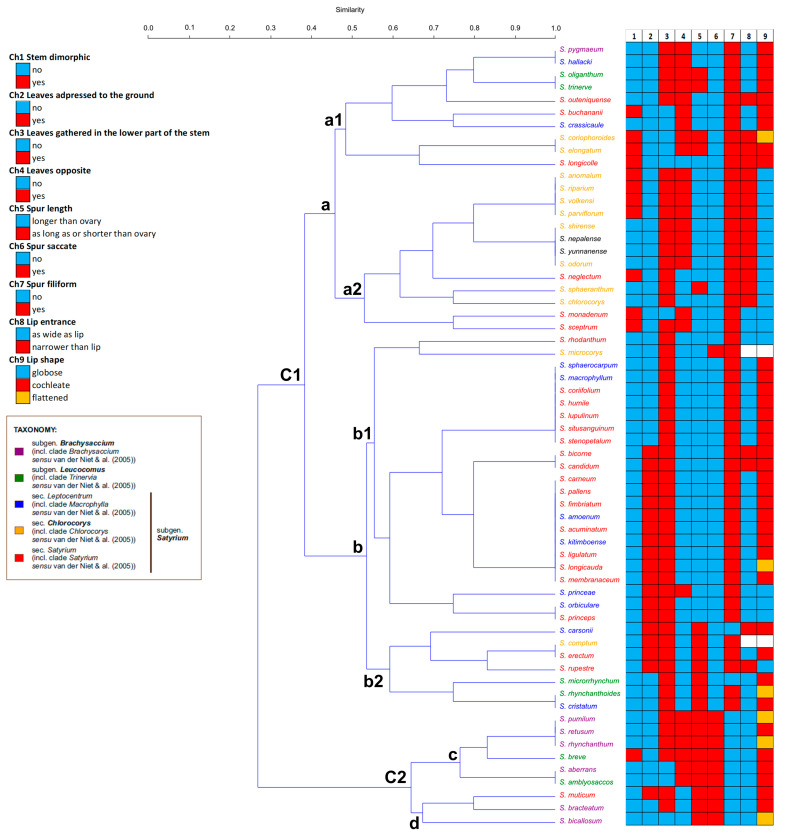
UPGMA cluster analysis based on Jaccard coefficients showing morphological similarities within the genus *Satyrium*. The taxonomic affiliation of each species has been included. A detailed description of the morphological characteristics can be found in Supplementary Table S2.

The resulting UPGMA dendrogram divided the specimens into two major clusters, which differed mainly in the trait describing the occurrence of saccate (Ch6) or filiform spurs (Ch7) (Figure 5). The first cluster (**C1**) included a greater number of species, from *S. pygmaeum* to *S. cristatum* and corresponded to species in which the filiform spur is not present, but instead is saccate. In contrast, the much smaller second cluster (**C2**) included species from *S. pumilum* to *S. bicallosum*, and the species grouped here have filiform spurs rather than saccate ones. The resulting subdivision and grouping of individual species did not reflect their taxonomic affiliation to the clades described in our phylogenetic analysis. Cluster (**C1**) was divided into two subclusters (**a** and **b**), which in turn were separated mainly on the basis of traits related to leaves adpressed to the ground (Ch2), opposite leaves (Ch4) and the entrance of the lip (Ch8). *Satyrium* species in subcluster **a** do not have leaves adpressed to the ground, but these leaves are opposite, and the lip entrance is narrow. In subcluster **b**, on the other hand, specimens mainly have leaves lying on the substrate, which are not opposite, and the lip entrance is rather wide. Subcluster **a** was further divided into two groups: **a1** included species from *S. pygmaeum* to *S. longicauda*, and **a2** included species from *S. anomalum* to *S. sceptrum*. This division is based on the Ch9 feature, where *Satyrium* species in group **a1** have a cochleate or flattened (like *S. coriophoroides*) lip, while in group **a2** the specimens have a globose lip. Group **a2** also included two Asian representatives of *Satyrium* (*S. nepalense* and *S. yunnanense*), which aggregate together with *S. shirense* and *S. odorum* from the *Chlorocorys* section. Subcluster **b** included species from *S. rhodanthum* to *S. cristatum* and was divided into two groups, **b1** and **b2,** based on the length of the spur (Ch5). Group **b1** (from *S. rhodanthum* to *S. princeps*) appears to be more consistent and is distinguished by a spur longer than the ovary, while group **b2** (from *S. carsonii* to *S. cristatum*) has a spur as long as or shorter than the ovary. It is worth mentioning that within group **b2**, the species assigned here are quite heterogeneous in terms of spur and lip shape (Ch7–9).

The second cluster (**C2**) revealed two smaller subclusters (**c** and **d**), comprising species that have leaves not adpressed to the ground (Ch2), a spur with a length equal to or shorter than the ovary (Ch5), a saccate spur (Ch6) and a wide lip entrance (Ch8).

Analysis conducted exclusively for characters associated with the spur and lip (Ch5–9) revealed five prevailing patterns of variation, but no better grouping of individual species reflecting their affiliation with the assigned sections was observed (Supplementary Figure S1). Again, the two Asian representatives of *Satyrium* (*S. nepalense* and *S. yunnanense*) are grouped together with species from the *Chlorocorys* clade.

**Figure 6:**
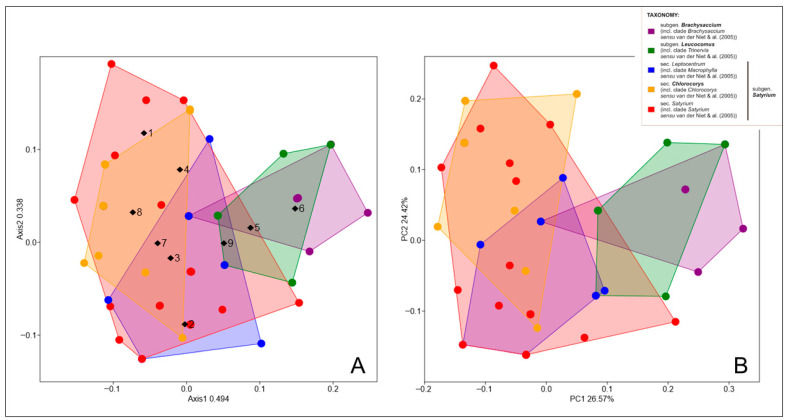
Nonmetric multidimensional scaling analysis (NMDS) (**A**) and principal coordinate analysis (PCoA) (**B**) showing the two-dimensional ordering of *Satyrium* species based on nine morphological characteristics. Characters (1–9 according to Table 1) were included in the NMDS plot as weighted averages of the row scores and are marked with purple dots. Convex hulls for each *Satyrium* clade were added to the plots.

NMDS and PCoA analyses showed that the division of sections among *Satyrium* was along the second axis (Figure 6). The cumulative percentage of variance explained by the first two axes in the PCoA analysis was almost 51%, while the stress value was S = 0.206, indicating an average match quality in NMDS. In both cases, representatives of *Trinervia* and *Brachysaccium* clades are grouped on the right side of the resulting scatter plots. This difference seems to be more related to flower morphology and specific floral patterns (see Figure 5) than to vegetative traits. However, the column scores in NMDS were consistent with the distinctive features for each section (Figure 6A). For example, the clades of *Trinervia*, *Leucocomus* and *Brachysaccium* are distinguished by three traits: a spur length equal to or shorter than that of the ovary (Ch5), the presence of a saccate spur (Ch6) and a cochleate or flattened lip (Ch9). In turn, SIMPER analysis indicated that lip shape (Ch9), lip entrance (Ch8) and the presence or absence of opposite leaves (Ch4) are the main factors responsible for the differences between *Satyrium* species (Table 1). However, the overall average dissimilarity was 49.62% for all the morphological characteristics used in our study.

### 2.4. Ancestral State Reconstruction of Morphological Features

Ancestral state reconstruction based on ITS and plastid trees (Supplementary Figures S2–S7) revealed extensive homoplasy in all morphological characters examined. Most of the reconstructed traits showed repeated and independent origins in different clades, followed by multiple losses, with essentially similar patterns in both datasets. For vegetative traits, the presence of leaves gathered at the base of the stem was confirmed for the most recent common ancestor (MRCA) of *Satyrium*, although this trait was subsequently lost several times within all major clades. Other leaf traits, such as opposite leaves or ground-adhering leaves, did not show consistent phylogenetic patterns and occurred sporadically across *Macrophylla*, *Chlorocorys*, *Trinervia* and *Satyrium* clades.

Floral traits showed a similar level of homoplasy. A cochleate lip was reconstructed for MRCA of the whole genus and remained common in various species, except for representatives of *Chlorocorys* clade, where it was lost independently. Globose lip or lip with a constricted entrance were not present in the *Brachysaccium* and *Trinervia* clades, but appeared independently in the *Satyrium*, *Chlorocorys* and *Macrophylla* clades.

The morphology of the spur also showed no phylogenetic consistency. A filiform spur was inferred for the MRCA of *Macrophylla*, *Chlorocorys*, *Satyrium* and *Trinervia* (plastid reconstruction only), but this trait showed several independent losses and gains within these groups. Saccate spurs were characteristic of the MRCA of *Brachysaccium*, although this state appeared sporadically also in the *Trinervia*, *Chlorocorys* and *Satyrium* clades. Spur length was also highly homoplastic within the clades.

Overall, the analyses revealed extensive parallelism and repeated state transitions across both species and higher-level lineages.

### 2.5. Biogeography

Reconstructions of ancestral geography, based on both nuclear and plastid data, show that regions of South Africa, mainly the Cape (A) and Natal (B), played a major role in the diversification of the genus *Satyrium* (Figure 2 and Figure 3; Supplementary Figures S8 and S9). This is consistent with the occurrence of most species of the genus today. The species known in this area are also highly diversified. Therefore, for the most part, it seems that the occurrence in southern Africa is an ancestral trait and that the diversification of most of the major *Satyrium* evolutionary lineages in the area took place in the early Miocene. On the other hand, the southern African regions have been occupied by different groups of species a few times in the geographic history of the genus. Species such as *S. cristatum*, *S. sphaerocarpum*, and *S. hallackii* also occupied this ancestral area but much later because of the Pleistocene (Figure 2 and Figure 3, Supplementary Figures S8 and S9).

Another very important region for the diversification of *Satyrium* is the mountainous area of eastern Africa (G), where at least a few lineages of the genus originated. However, in the case of this region, this occurred later, most likely at the end of the Miocene and the beginning of the Pliocene. The East African mountain area also played an important role as a place where both the recolonisation of southern African areas and new areas took place. These include the Malagasy taxa of *Satyrium*, here represented by *S. amoenum*. Analysing reconstructions of ancestral distributions, it can be assumed that the common ancestor occurred both in Madagascar (L) and in the East African Mountains (G) regions from which the island was colonised, probably in the middle Miocene. A similar situation may have occurred for Asian (M) species, but colonisation of the continent occurred later, in the late Miocene and through different lineages, but again, most likely from the mountainous areas of eastern Africa.

Finally, colonisation by *Satyrium* taxa of the Zambezian (C, D, E, F), Guinea-Congolan (H), Sudanian (I), Kenyan (J), and Ethiopian Highlands (K) regions occurred independently in several lineages and usually took place relatively contemporaneously in the early Pliocene (Figure 2 and Figure 3, Supplementary Figures S8 and S9).

Although the matrices based on nuclear and plastid data are not congruent, the reconstruction of ancestral ranges is consistent between them. Both indicate the Cape region (A) as a key area for the diversification of most taxa. A certain difference appears in the nuclear matrix, where the Natal region (B) is also inferred together with the Cape region at many nodes. A similar pattern is observed for mountainous areas (G), which appear as important in the evolution of *Satyrium*, including the migration of the genus, in both matrices. However, due to differences in clade resolution, this region is more frequently inferred together with the Cape region (A) in the nuclear matrix. In summary, any differences between the matrices result from different resolutions of some clades and from the differing positions of individual taxa. However, the reconstruction of regions itself retains the same main pattern across the matrices.

## 3. Discussion

### 3.1. Morphological Variation and Divergence

The *Satyrium* genus exhibits considerable morphological diversity, both in terms of vegetative and floral characteristics, a pattern commonly observed in Orchidaceae, although usually in much larger genera, such as the African *Disa* [26] or the Neotropical *Oncidium* Sw., *s.l.* [27], *Maxillaria* Ruiz & Pav., *s.l.* [28], *Encyclia* Hook. [29], and *Prosthechea* Knowles & Westc. [30]. This diversity is influenced by various factors, including pollinator interactions, habitat preferences and biogeographical history. However, morphological variation often does not correspond to phylogenetic relationships, which is often due to homoplasy. In the case of *Satyrium*, the occurrence of similar traits in different evolutionary lineages is likely due to divergence rather than convergence [26,31]. These lineages began to diverge at the end of the early Miocene and in the late Miocene, and the development of similar traits was driven by similar environmental pressures.

We analysed traits related to two types of stems, sterile and flowering stems, as well as leaf distribution along the stem. Some of these traits appeared in multiple evolutionary lineages, especially dimorphic stems, which developed in two groups, the *Satyrium* and *Chlorocorys* clades, mainly in the mountainous regions of Eastern Africa. This trait may be linked to the plants’ lower tolerance to low temperatures [32] and the adaptive strategy of producing lateral flowers in order to survive in harsh conditions [33,34]. Similar adaptations are observed in other orchid genera, such as *Habenaria* Willd. *s.l.*, *Holothrix* Rich. *ex* Lindl., and *Brachycorythis* Lindl. *s.l.* In contrast, the basal leaf arrangement in *Satyrium*, which is probably a plesiomorphic feature, suggests an adaptation to arid, rocky habitats. Stronger adaptations, like leaves adhering to the ground, evolved independently in several lineages, particularly in species from southern Africa and mountain regions [35].

Morphological diversity, arising from convergent evolution, can lead to erroneous conclusions about species relationships and result in polyphyletic taxa. As suggested in previous studies, *Satyrium*’s floral diversity is linked to a range of pollinators [36,37], similar to *Disa* species [38]. Johnson et al. [37] identified four main flower types in the genus *Satyrium*. The first, with long, filiform spurs, attracts moths; the second one, with slender, cylindrical spurs, is bird-pollinated; the third one, with long, cylindrical spurs, is bee-pollinated; and the fourth, with short, saccate spurs, is fly-pollinated. Several other features, such as the form and size of viscidia and apical flaps, correlate with specific types of spurs. Our results indicate that traits such as lip shape, entrance width, and spur shape and length have developed in diverse ways in different groups. The cochleate lip is likely a plesiomorphic trait, with an undefined spur structure. *Satyrium* species exhibit a wide range of pollinators, which influences their floral structure. These pollinators include moths, beetles, wasps, bees, and birds [39,40,41,42,43]. Therefore, the wide variety of floral adaptations in many evolutionary lineages is probably the result of pollinator-driven selection. This phenomenon has been extensively studied in *Disa* species by Johnson et al. [38], who showed that the adaptive radiation of these orchids is closely linked to the evolution of a unique pollination system.

### 3.2. Phylogeny Incongruence

Recent advances in nuclear genome sequencing have highlighted discrepancies in phylogenetic trees constructed from different genetic markers, often caused by hybridization or incomplete lineage sorting (ILS) [44]. ILS occurs when alleles at multiple loci segregate randomly over short evolutionary periods, leading to discordant tree topologies. Maddison [45] introduced the method of minimising deep coalescences (MDC) for species tree reconstruction, assuming that ILS was the main cause of these discrepancies. Hybridization and ILS can be distinguished: ILS is a stochastic process, while hybridization is a directional phenomenon and reduces genetic divergence at specific loci. However, distinguishing between the two mechanisms requires the analysis of multiple loci [46]. Due to the reticulate evolutionary history of many lineages, modern phylogenetic methods have been developed to consider both ILS and hybridization simultaneously [47,48]. The phylogenetic network (Figure 4) reveals two main patterns of conflict between plastid and nuclear datasets. The first concerns individual species that occupy discordant positions in different data partitions. The second type of conflict concerns broader clades, in particular the Asian group of species (*S. nepalense*, *S. ciliatum*, and *S. yunnanense*) and Clade III. These patterns indicate a complex evolutionary history within the genus *Satyrium*. However, given the limited set of available markers, it is not possible to determine whether these conflicts result from incomplete lineage sorting, hybridization or other processes. Nevertheless, in the case of the Asian species group, additional information already available from previous studies may further confirm the possibility of hybridization.

Hybridization is common in plants, particularly in the Orchidaceae family [47,48], and the genus *Satyrium* is no exception. Conflicts between nuclear- and plastid-based phylogenies suggesting hybridization have been previously reported [1]. Phylogenetic incongruence interpreted as evidence of hybridization has been repeatedly documented in *Satyrium* [1,49,50], with eleven hybrid combinations involving 13 species identified [51].

Based on the observed difference in chromosome numbers between African (n = 21) and Asian (n = 41) *Satyrium* species [52], we propose that the ancestor of the Asian lineage originated in Africa through allopolyploidization, followed by chromosome-number reduction via descending dysploidy—a process often associated with post-polyploid diploidization in plants [53,54,55]. Such polyploid species can occupy new ecological niches and may have subsequently dispersed from Africa to Asia [56]. Although the specific genomic contributions of diploid ancestors remain uncertain, studies on *Dactylorhiza* indicate that allotetraploid orchids often exhibit mixed inheritance due to genome heterogeneity and recombination [57,58].

This scenario remains hypothetical and requires further verification through detailed cytogenomic analyses, including chromosome-level comparisons and genomic sequencing, to confirm the polyploid origin and subsequent chromosomal evolution of the Asian *Satyrium* lineage.

### 3.3. Biogeographical Factor of Evolution

Reconstruction of ancestral geographical ranges in *Satyrium* has shown that the regions of South Africa and the mountainous area of Eastern Africa played the most important role in the diversification of the genus. The differentiation of most evolutionary lineages took place from the end of the early Miocene through the late Miocene and into the Pliocene (Figure 2 and Figure 3).

During the early Miocene, the evolutionary lines of the major clades of *Satyrium* diversified. Most likely, two of the most crucial periods were involved in the evolution of the group during this time. The Miocene Climatic Optimum (MCO) ended ca. 14.7 Ma, and the climate temperature began to gradually decline [59,60,61,62]. That is, the Miocene Climate Transition (MCT) period commenced, lasting until ca. 13 Ma [59,63]. It is suggested that during this period, there was a reduction in forest areas in Africa in favour of open and grassy lands [62], which provided an opportunity to diversify a number of species of dry-adapted plants, such as *Satyrium*. Other important events of the early Miocene include the orogenesis phenomena occurring in eastern Africa, although the surface of Africa was still at a lower elevation than it is today [62,64,65].

The late Miocene and mid-Pliocene were characterized by further cooling of the climate, with a more rapid decline in temperature between the Late Miocene Cooling (LMC) period and ca. 7 and 5.4 Ma [59]. This led to a significant climatic gradient in Africa, which was consistent with latitude. The consequences of this were further aridification of some areas, such as southern Africa, and an increase in the proportion of open and grassy lands. Important events affecting the increase in plant diversity in Africa were associated with the uplift of Ruwenzori, Mt. Kenya, or the formation of Lake Malawi, which increased topographical complexity [62,66]. This was also the case when the greatest differentiation of *Satyrium* occurred.

It appears that the mountainous region of eastern Africa, which is also a contact zone where different lineages could have potential opportunities for hybridization, played an important role in the evolution of *Satyrium*. Analysis of the reconstruction of geographical distribution showed that hybrids originated between several evolutionary lines in the region of East Africa, and some of the most significant hybrids were between Asian species (Figure 2 and Figure 3). The subsequent colonization of Asia must have occurred from this region. It remains unexplained how the *Satyrium* reached Asia. Although the literature is dominated by long-distance transport theory [67,68,69,70,71,72], it has also been suggested that migration may have been overland [73,74,75,76] and that the occurrence of *S. brachypetalum* A.Rich. in Yemen may support this hypothesis. In both *S. brachypetalum* and *S. nepalense s.l.*, the inflorescence is slender, many-flowered, the floral bracts are reflexed, the spurs are equal in length to the ovary, and the lip entrance is relatively small. However, *S*. *brachypetalum* has two types of stems, sterile and fertile, while *S*. *nepalense* has only one. Given the late Miocene age (ca. 6 Mya) of the Afro–Asian disjunction, long-distance dispersal mediated by wind currents represents the most parsimonious explanation [70,71]. By this time, progressive aridification of the Arabian Peninsula likely precluded the existence of a continuous terrestrial migration corridor, whereas established monsoonal circulation systems could have facilitated rare but effective transoceanic dispersal events.

The current distribution of species largely reflects phylogenetic relationships within the genus. It can therefore be assumed that geographic history has played a major role in shaping its taxonomic diversity.

### 3.4. Infrageneric Classification

The classification of organisms is complex and involves both phylogenetic relationships and morphological features that should be considered when distinguishing taxa [77]. Phylogenetic studies often lead to the creation of large, difficult-to-define taxa or very narrow groups. Hybridization further complicates this task by allowing gene flow between evolutionary lineages, creating a mosaic of morphological traits. Studies have shown that a significant proportion of vascular plant species have undergone hybridization events [54,78,79], and in the case of *Satyrium*, 29.4% of species are involved in hybrid formation [51]. Morphological traits such as spur length, lip entrance, leaf position on the shoot, and shoot biformity also exhibit plasticity in response to environmental conditions, leading to convergences in distant evolutionary lineages.

Intrageneric classification is often based on a few morphological traits to separate taxa, resulting in non-monophyletic groups. On the other hand, one must ask whether the infrageneric division must necessarily reflect phylogenetic relationships or whether it should be practical and make it easier for botanists to navigate within multiple species and morphologically diverse genera. Although phylogenetic studies have revealed that traditional classifications of *Satyrium* are not consistent with its evolutionary history, distinguishing clear apomorphies for each clade has proven difficult. Although some features that define these clades have been identified [1], few of them are non-homoplasious.

Asian *Satyrium* species have not yet been classified into intraspecific units, due to their hybrid origin, which presents a challenge to genomic data. However, morphological similarities, such as leaf shape and arrangement, lip shape or spur shape and size, support the inclusion of these taxa within the *Chlorocorys* section. This would reflect both their morphological resemblance and their evolutionary history.

In the basal lineage of *Satyrium*, flowers evolved with myophyllous traits, while other groups adapted to pollination by hymenoptera. Most *Satyrium* species are pollinated by moths, butterflies, birds, and bees. In the latter group, two distinct morphological lines can be distinguished: one with obstructed access to the reproductive structures through the narrow entrance to the lip and the other with exposure of the gynostemium, not restricted by the entrance to the lip. The latter feature appears to be a plesiomorphic character state, as it occurs in different phylogenetic lineages, namely, *Leptocentrum* and *Satyrium*. At this stage of our research, we are unable to identify the key morphological features that distinguish them and would be stable across all species. Studies by Johnson et al. [37] suggest that gynostemium traits, particularly the rostellum and viscidial structures, might offer insights into these differences. Unfortunately, we did not have access to well-preserved material from the species studied here. Indeed, herbarium specimens are not always suitable for the investigation of delicate structures such as gynostemia.

### 3.5. Taxonomic Treatment

We propose to recognize three groups within the genus *Satyrium* at the subgeneric rank, which are correlated with adaptation to different pollinators. Unfortunately, not all type species in the taxonomic units were included in the analyses (subgen. *Leucocomus* and sect. *Leptocentrum*). Therefore, based on the other taxa, the proposed groups are expected to be accurate; however, they should be approached with caution and subjected to validation through expanded sampling.

Furthermore, in distinguishing the sections *Satyrium* and *Leptocentrum*, we used leaf arrangement characteristics. However, we must emphasize that although leaves adhering to the ground are traditionally cited as a feature of the *Satyrium* section, this trait is sometimes lost in certain representatives of this group and can also appear in some representatives of *Leptocentrum*. The structure of the rostellum and viscidium within these sections should be verified, as they have the greatest potential for distinguishing them.

Key to the infrageneric groups:

1. Spur more or less saccate—subgen. *Brachysaccium.*

1.* Spur never saccate—2.

2. Spur short and cylindrical—subgen. *Leucocomus.*

2.* Spur long and filiform—3. (subgen. *Satyrium*).

3. Lip with narrow entrance—sect. *Chlorocorys.*

3.* Lip with wide entrance—4.

4. Leaves usually adpressed to the ground—sect. *Satyrium.*

4.* Leaves usually not adpressed to the ground—sect. *Leptocentrum.*

(1) Subgenus *Brachysaccium* (Schltr.) Kurzweil & Linder

This is the most basal evolutionary line of *Satyrium*. It can be characterized by short, more or less saccate spurs. The entrance to the lip is very wide, which makes the generative structures of the plant easily accessible to insects. The lip is usually cochleate, but in some cases, a more or less flattened lip can be observed. The flowers are either greenish, brownish or a combination of both colours; not pleasantly fragrant with a carrion smell; or white or off-white with a sweetish fragrance. All these characteristics indicate adaptation to pollination by flies. Floral bracts are relatively wide-spreading. Leaves gather in the lower part of the stem. Species in this group are most common below 2000 m above sea level.

(2) Subgenus *Leucocomus* (Schltr.) Szlach.

Clade *Trinervia sensu* van der Niet & et al. [1]

The species of this group have cochleate lips combined with rather short and cylindrical spurs. The flowers are usually white or pinkish and are rarely greenish. The lip is cochleate, except for *S. rhynchanthoides*, in which the lip is flattened; hence, the gynostemium is easily accessible to potential pollinators. The species are likely to be adapted for pollination by bees or flies. Floral bracts are lanceolate and usually spreading. Leaves gather in the lower part of the stem, but dimorphic stems can be observed in *S. breve*. Most species grow between 1000 and 2300 m above sea level.

(3) Subgenus *Satyrium*

It includes species (incl. type species *S. bicorne*) with a filiform spur combined with a cochleate lip in the majority of species, but with some exceptions having a globose lip. The latter character evolved independently in a few lines of the subgenus. The reproductive structures are easily accessible. In the group of species included in the section *Satyrium*, the flowers are mainly white, less frequently yellow or pink. Most species occur at lower altitudes, usually below 1200 m. Among the species in the “*Leptocentrum*” section, flower colours are more varied, ranging from red and purple to white and greenish. Most of the species inhabit areas at an altitude of 1000–2000 m. As the flowers exude a pleasant fragrance and have long spurs, it can be assumed that they are moth- or butterfly-pollinated. In a group of species previously classified in the section “*Leptocentrum*” (incl. type species *S. longicauda*), the stem is dimorphic. In all other species of the subgenus, the stem is not biformed. It appears to have been beneficial to retain the leaves lying on the substrate due to the habitat occupied. The species occupying a basal position to all other subgenus representatives is *S. lupulinum*.

(3.1) Section *Satyrium*

It includes species adapted to pollination by animals with long apparatuses. The plesiomorphic state is a wide entrance to the lip.

(3.2) Section *Leptocentrum* Schltr

Clade *Macrophylla sensu* van der Niet & et al. [1].

Species of this group are characterized by a cochleate lip, with the exception of *S. carsonii*, in which the lip is globose. The lip entrance is wide; hence, the reproductive structures are exposed. The only exception is *S. carsonii*, in which the entrance is somewhat squeezed. The spurs are long and filiform. The flowers are usually white, pink or a combination of both of these colours; rarely, they are orange or purple. They can be pollinated by butterflies, moths or birds. Considering the foliage, in some species, the leaves lie on the ground (*S. kitimboense*, *S. amoenum*); in others, they are gathered in the lower part of the stem. Most species are found at altitudes of 1000–2000 m

(3.3) Section *Chlorocorys* Schltr.

The group includes species classified in the former section, *Chlorocorys* (incl. type species *S. chlorocorys*). It can be diagnosed by a globose lip with a very narrow entrance, a characteristic that is lost in some species (*S. microcorys* and *S. comptum*). In all species, the spur is filiform. The colour of the flowers varies; most often, they are white or less pink-tinged, and often they are pink or yellow, sometimes with a green tint. Modest information indicates that the flowers emit a pleasant fragrance. All these characteristics can be interpreted as adaptations to pollination by insects with elongated apparatuses. Narrow lip entrances can suggest moth pollination. In a large group of species, the stem is dimorphic. Leaves gather in the lower part of the stem. The African representatives of the group usually grow between 1000 and 3000 m above sea level, and the Asian species can be found at altitudes of 2000–3700 m.

## 4. Materials and Methods

### 4.1. Phylogenetic Analyses

Our analyses were based on 68 *Satyrium* species, representing approximately 75% of all species described within the genus. Sampled taxa represent all major clades, providing a comprehensive overview of the phylogenetic diversity of the genus. The datasets also included representatives of related genera (*Disa* and *Corycium*). *Disperis capensis* Sw. was selected as an outgroup. We used sequences of the nuclear internal transcribed spacer (ITS), plastid *matK*, *trnS*-*trnG* intergeneric spacer, *trnL*-*trnF* intergeneric spacer and *trnL* intron, available in GenBank (all accession numbers are given in Supplementary Table S1). We prepared two separate matrices—nuclear (85 specimens; 699 bp) and plastid (80 specimens; 3100 bp) markers. MAFFT v.7. [80] was used for sequence alignment. All minor errors were then corrected in SeaView v.5.0. [81].

Nucleotide substitution models were calculated separately for each matrix using AIC (Akaike Information Criterion) on the PhyML website “http://www.atgc-montpellier.fr (accessed on 22 May 2025). The following models were selected as the best fitting: GTR + G (nuclear dataset) and GTR + G + I (plastid dataset).

To confirm the incongruence between the nuclear and plastid datasets, an incongruence length difference test (ILD) [82] was performed in PAUP v4.0b10. [83]. The analysis (with 100 replicates and random addition sequences and tree-bisection-reconnection—TBR) yielded *p* values lower than 0.01, indicating significant incongruence [84]. Therefore, we decided not to perform a combined analysis.

The divergence time reconstruction was performed in BEAST v.1.8.4. [85], on the CIPRES Science Gateway platform [86]. The age of the tree root was chosen according to results presented by Givnish et al. [87], where the corresponding node was dated to 38.1 Mya. The stepping-stone/path-sampling methods were implemented to calculate the Bayes factor (2log_e_(BF)) and define the type of molecular clock [88]. The results indicated that the relaxed molecular clock was more probable for both studied datasets. A lognormal distribution and Yule model of speciation were used. The analyses were performed in two independent runs, each with 40,000,000 generations. Tracer v. 1.6. [89] was used to verify the quality of the results, and then the .log files were combined in LogCombiner v. 1.8.4 [85]. Additionally, a maximum likelihood (ML) analysis was performed in raxmlGUI 2.0 [90], with 1000 bootstrap replicates for each of the studied datasets. The posterior probability (PP) values from Bayesian inference (BI) and the bootstrap support (BS) values from maximum likelihood (ML) reconstructions are given as PP/BS, only if they reached values above 0.5 (PP) and 50 (BS support). According to Pelser et al. [91], PP ≥ 0.95 and BS ≥ 80 were considered reliable.

To demonstrate potential hybridization events in *Satyrium*, the autumn algorithm method [92] with default options (edge weighting = equal, threshold = automatic) was used to construct a hybridization network in SplitsTree6 [93]. The hybridization network was based on two incongruent Bayesian trees (ITS and plastid), where nodes supported lower than 0.98 PP were removed, thus keeping nodes BS ≥ 80. This approach highlights patterns of topological conflicts between datasets and allows to identify branches whose reticulation may be consistent with processes such as hybridization or incomplete lineage sorting.

### 4.2. Morphological Analyses

The study incorporates morphological characteristics of supraspecific taxonomic relevance, with particular attention to ensuring consistent definition and coding of traits. Morphological data were obtained through an extensive survey of approximately 1500 herbarium specimens from the following collections: AMES, BM, BR, EA, G, K, MO, P, W, WRSL, UGDA, US and Z. To reduce subjectivity and improve analytical depth, each character was defined based on discrete, repeatable states observed across specimens. Although most traits were coded as qualitative variables, multistate character states were applied wherever variation exceeded a simple binary distinction. The rationale for character circumscription and coding strategy is provided in Supplementary Table S2.

For the qualitative morphological analyses, 62 *Satyrium* species were described using the nine most discriminating traits. The taxonomically significant features were considered to be those indicated by previous researchers who either published partial revisions of the genus or considered phylogeny in the systematics of the genus with discussion about morphology [1,6,7,8,10,11]. Also, it is indicated by our preliminary research towards taxonomic revision indicates that the features we have chosen are relevant. The selected traits referred to apparent external differences for stem and leaves (Ch1–4) and for floral characters related to spur and lip structure (Ch5–9). To investigate morphological variation, the mentioned characters were binary coded <0,1> and were often opposite in nature, except for the trait describing lip shape, Ch9, which was included in the three states of this trait (Supplementary Table S2).

The data prepared in this way were applied to a hierarchical cluster analysis using the unweighted pairwise groups with arithmetic average (UPGMA) method (Supplementary Table S3). The similarity between taxa was calculated using the Jaccard similarity coefficient. The same analysis was also carried out based on floral traits only (i.e., Ch5–9).

In addition, to assess morphological variation among *Satyrium* sections, we performed principal coordinate analysis (PCoA) and nonmetric multidimensional scaling (NMDS), both based on the Jaccard similarity matrix. PCoA emphasizes linear relationships among samples, whereas NMDS captures rank-order similarities. To identify traits contributing most to morphological differentiation, a SIMPER analysis was conducted using the Bray–Curtis dissimilarity matrix. The average contributions of each character to group differences are summarized in Supplementary Table S4 in the context of the taxonomic subdivisions inferred from our phylogenetic analysis.

All multivariate analyses and tests were performed using the software packages STATISTICA v. 13 [94] and PAST v. 4.14 [95]. Moreover, the ancestral states of the same morphological traits were reconstructed using Mesquite software v.3.81 [96]. Due to extensive topological conflicts within the studied group, the analyses were conducted separately for the ITS and plastid datasets with the parsimony ancestral state reconstruction method. Our phylogenetic trees were used from molecular clock analyses as the tree input files.

### 4.3. Biogeography

Ancestral geographical range in *Satyrium* was reconstructed in RASP 4.3 [97]. Due to the incongruence of trees based on nuclear and plastid markers, the analyses were conducted separately for both datasets. Model selection was tested between the DEC [98], DIVALIKE [99], and BAYAREALIKE models [100], and initially included the ‘J’ parameter for each of them [101]. The models were ranked according to corrected Akaike information criterion (AICc) and the BAYAREALIKE model, featuring the lowest AICc value, was considered the ‘best’ for both nuclear and plastids data. Due to the large number of adopted regions, the BayArea analysis was finally conducted, as it is recommended as the solution in this case [100]. The preference for the BayArea model is biologically plausible given the high dispersal potential of orchid seeds and the lack of clear vicariant events structuring the distribution of *Satyrium*. In all analyses, the maximum clade credibility trees were used for reconstruction. Since it is recommended to test whether the obtained model is stable and the results are consistent in the case of the Bayesian approach to biogeographic inference [101,102], the results were tested using R v.4.3.1 [103] and the coda package [104]. Finally, the chains were run for 6 million generations, sampling every 1000 of them, and burn-in set to 0.25 (Supplementary Materials S1 and S2).

Eleven biogeographic regions have been defined based on floristic regions delimited for continental Africa [105], with some modifications based on Linder et al. [106] and Droissart et al. [107] with respect to the occurrence pattern of *Satyrium* species. These include areas in southern Africa adopted after Linder et al. [106] and mountainous regions in eastern Africa considered here together, but recognized by Droissart et al. [107]. The use of a biogeographic division based on species distributions and the physical environment [105] made it possible to avoid an artificial division into regions. In the case of Satyrium, where many species are endemics or plants adapted to very specific conditions, this approach provided a reliable picture of the distributional history of the genus. Areas hatched by the pattern were not included as relevant in the analysis of the distribution of *Satyrium*, and even if single locations were in them, we assumed the region of the most frequent occurrence of the species. Finally, we identified the Cape (A), Natal (B), Zambezian-East Coast (C), Zambezian-South (D), Zambezian-Central (E), Zambesian-West (F), East African Mountains (G), Guinea-Congolian (H), Sudanian (I), Kenyan (J), and Ethiopian Highlands (K) regions (maps on Figure 2 and Figure 3). Another two separate regions are Madagascar (L) and Asia as a whole (M).

## 5. Conclusions

The genus *Satyrium* is characterized by high species richness and morphological variability. Understanding such taxa is greatly facilitated by predictive infrageneric classification, which was the main goal of our work, using molecular, morphological and geographic data. The observed multiple gene flows between different evolutionary lineages, adaptations to specific habitat conditions, and independent evolution of pollinators in different groups obscure the pattern of phylogenetic relationships, making intrageneric classification of *Satyrium* difficult and forcing the search for a compromise between clades and grades. The analyses carried out in this article suggest that the key feature in the evolution and thus the classification of *Satyrium* species is their adaptation to different groups of pollinators. The phenomenon of hybridization within *Satyrium* at different times during the evolution of the genus means that most infrageneric divisions are in conflict with phylogeny based on a single marker. However, using a dated phylogeny as a starting point but supported by morphological and geographic data provides an opportunity to propose a well-justified classification. Nevertheless, given the substantial limitations of the present study, the proposed scenario should be interpreted with caution, and further research aimed at reducing these limitations is clearly needed. The most critical issue concerns the phylogenetic resolution of Satyrium, as several nodes currently lack sufficient statistical support. Genomic-scale data, therefore, represent a particularly promising avenue for future research. Additionally, incongruence between the nuclear and plastid datasets was detected. Although hybridization may represent a major driver of this conflict, alternative evolutionary processes could generate similar patterns and should be explicitly tested in future studies. Finally, although pollinators are identified here as one of the key factors shaping diversification within the genus, considerable gaps in empirical knowledge remain in this area. At present, the proposed scenario should be regarded as a working hypothesis, reflecting a synthesis of the available evidence rather than a definitive reconstruction of the evolutionary history of *Satyrium*.

## Figures and Tables

**Table 1:** SIMPER analysis results showing the contribution of traits influencing *Satyrium* species grouping. Overall average dissimilarity = 49.62%. For a detailed description of the morphological characters, see Supplementary Table S2 (Av. dissim.: average dissimilarity; Contrib. %: percentage of dissimilarity explained by individual traits; Cumulative %: cumulative percentage of Bray–Curtis similarity).

Character	Av. Dissim.	Contrib. %	Cumulative %
**9** Lip shape	10.67	21.51	21.51
**8** Lip entrance	8.80	17.74	39.25
**4** Leaves opposite	6.14	12.37	51.62
**5** Spur length	5.60	11.29	62.91
**2** Leaves adpressed to the ground	4.70	9.47	72.38
**7** Spur filiform	3.96	7.99	80.37
**1** Stem dimorphic	3.48	7.02	87.39
**6** Spur saccate	3.32	6.70	94.09
**3** Leaves gathered in the lower part of the stem	2.93	5.91	100

## Data Availability

The original contributions presented in this study are included in the article/Supplementary Materials. Further inquiries can be directed to the corresponding author.

## Supplementary Materials

The following supporting information can be downloaded at: https://www.mdpi.com/article/10.3390/ijms27010453/s1.
