# Supplementary material for: A Multi-Evidence Approach to the Systematics of the Genus Satyrium Sw. Based on Time-Calibrated Phylogeny, Morphology, and Biogeography
Source: Int J Mol Sci. 2025 Dec 31;27(1):453. doi: 10.3390/ijms27010453 (PMC12787166; doi:10.3390/ijms27010453)

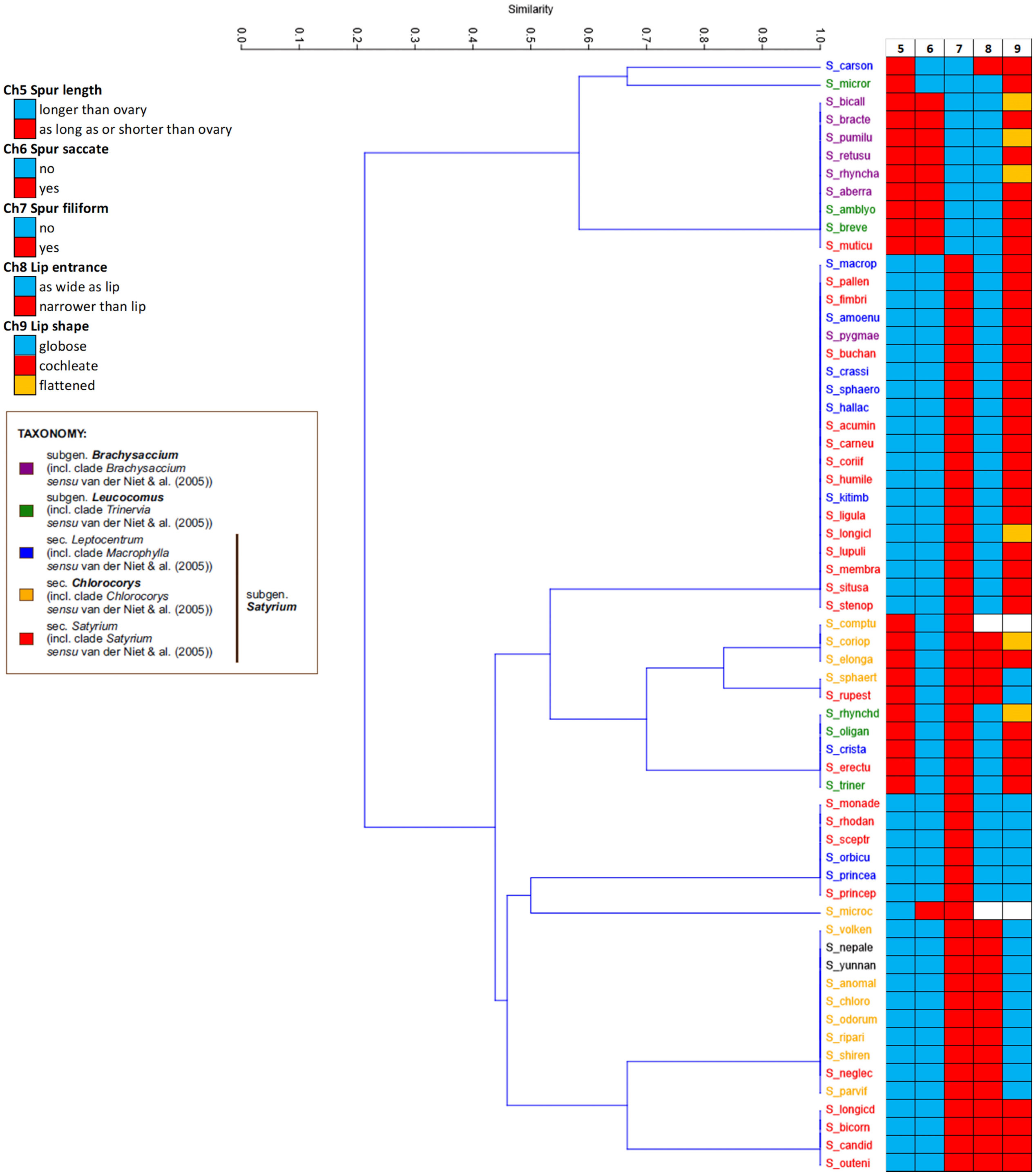

Fig. S1. Two-way UPGMA cluster analysis based on Jaccard similarity coefficients, showing morphological subdivision between *Satryrium* species, in relations to their taxonomic affiliation. A detailes description of the morphological characteristic can be found in Supplementary Table S2.

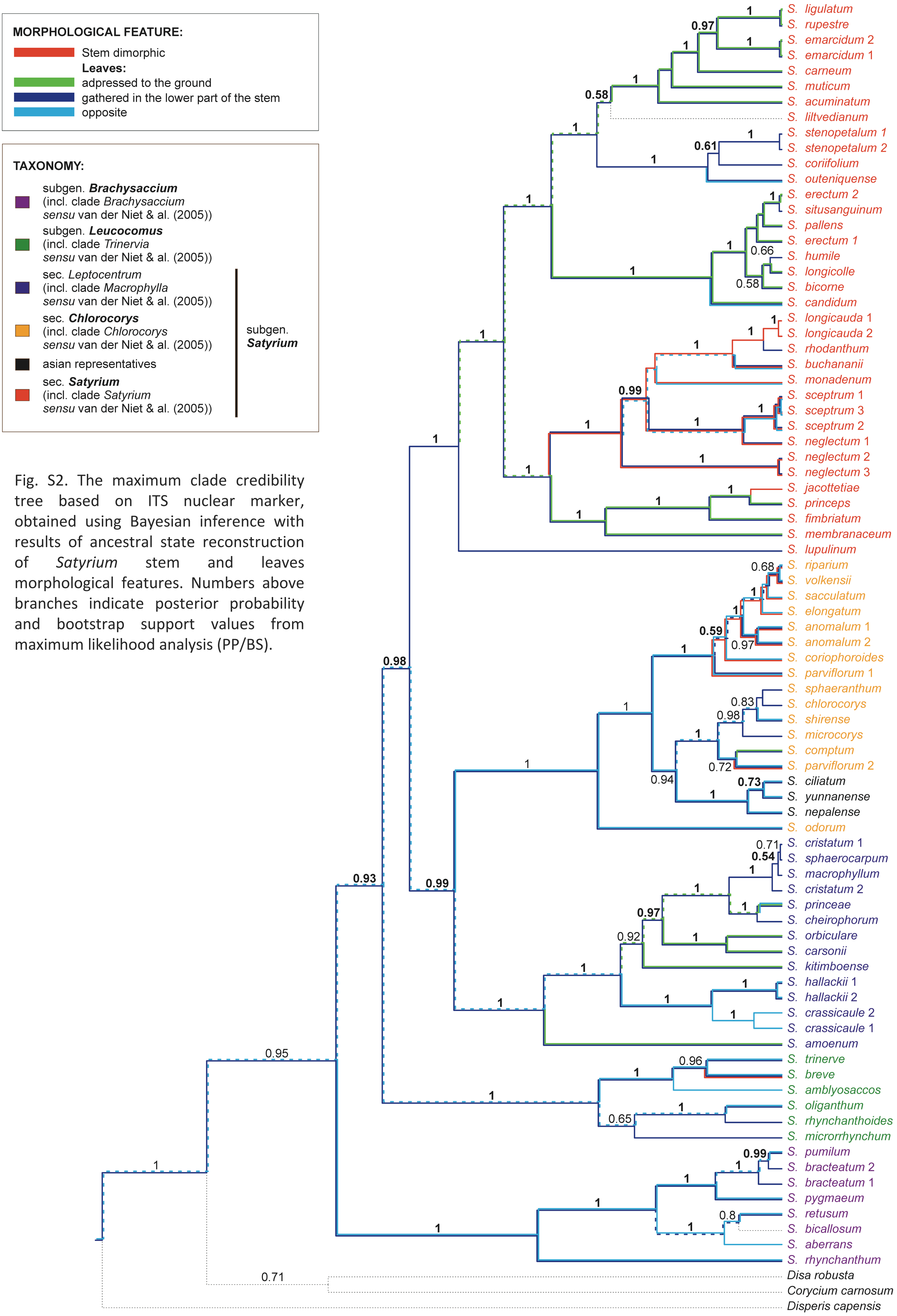

Fig. S2. The maximum clade credibility tree based on ITS nuclear marker, obtained using Bayesian inference with results of ancestral state reconstruction of *Satyrium* stem and leaves morphological features. Numbers above branches indicate posterior probability and bootstrap support values from maximum likelihood analysis (PP/BS).

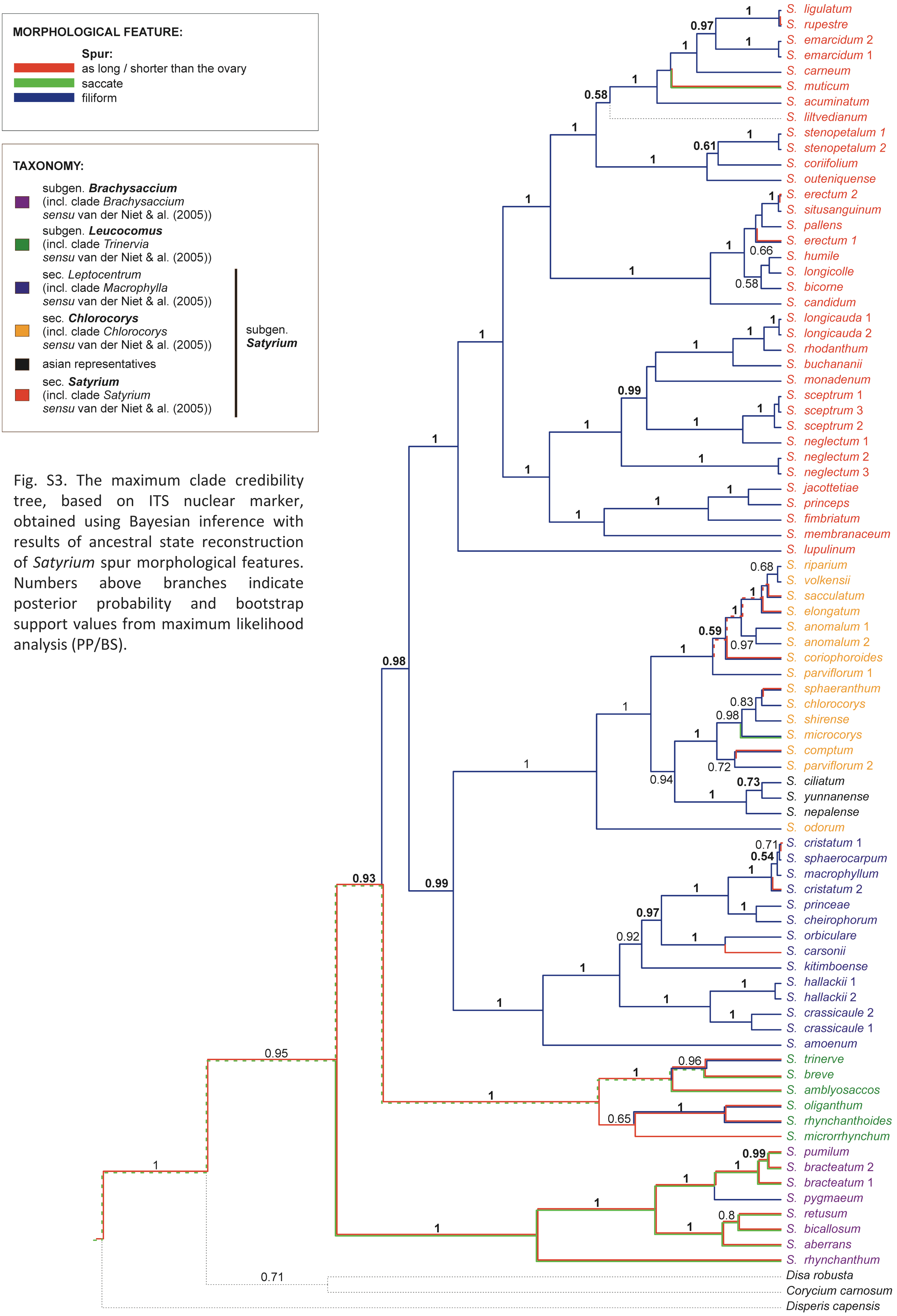

Fig. S3. The maximum clade credibility tree, based on ITS nuclear marker, obtained using Bayesian inference with results of ancestral state reconstruction of *Satyrium* spur morphological features. Numbers above branches indicate posterior probability and bootstrap support values from maximum likelihood analysis (PP/BS).

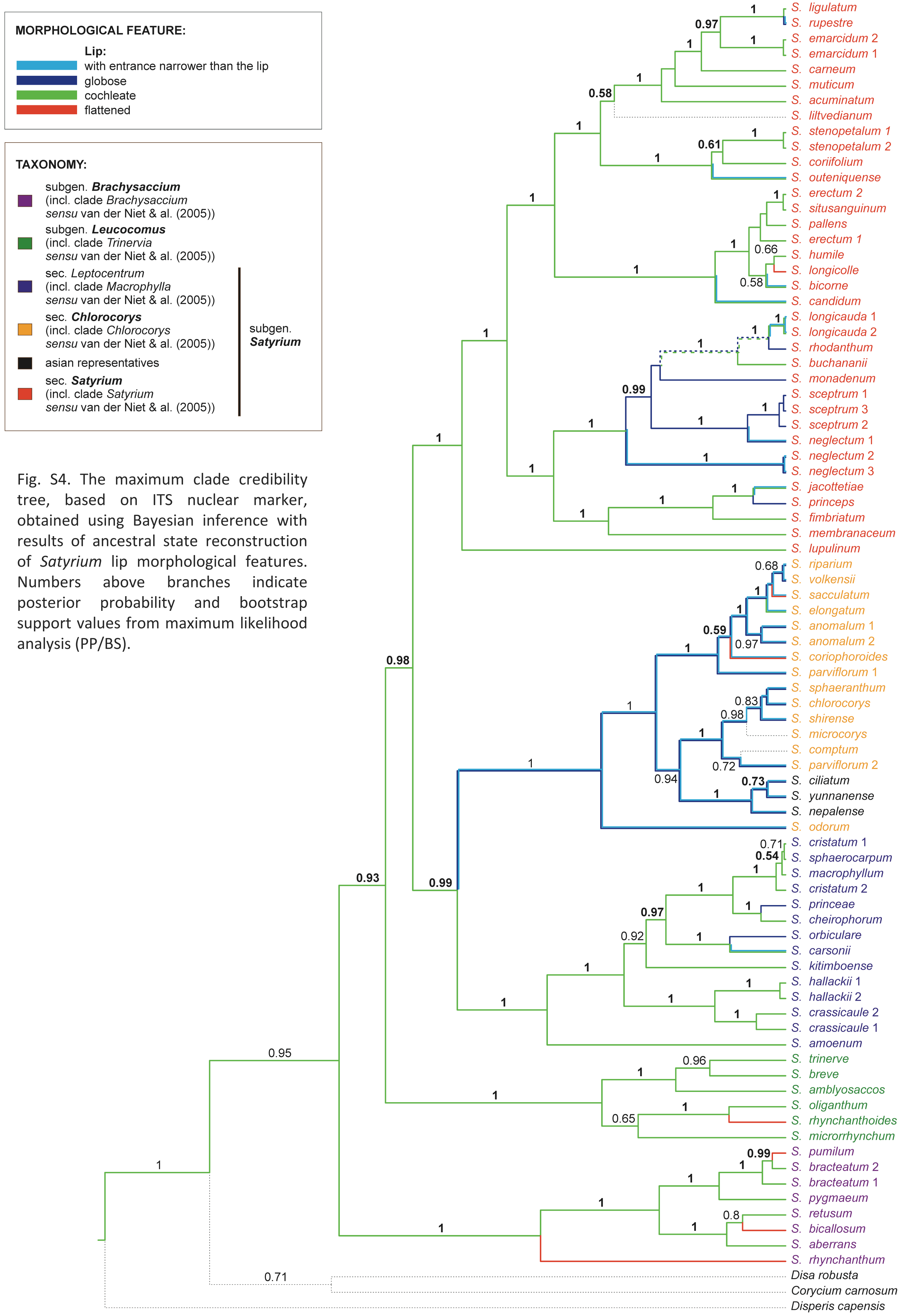

Fig. S4. The maximum clade credibility tree, based on ITS nuclear marker, obtained using Bayesian inference with results of ancestral state reconstruction of *Satyrium* lip morphological features. Numbers above branches indicate posterior probability and bootstrap support values from maximum likelihood analysis (PP/BS).

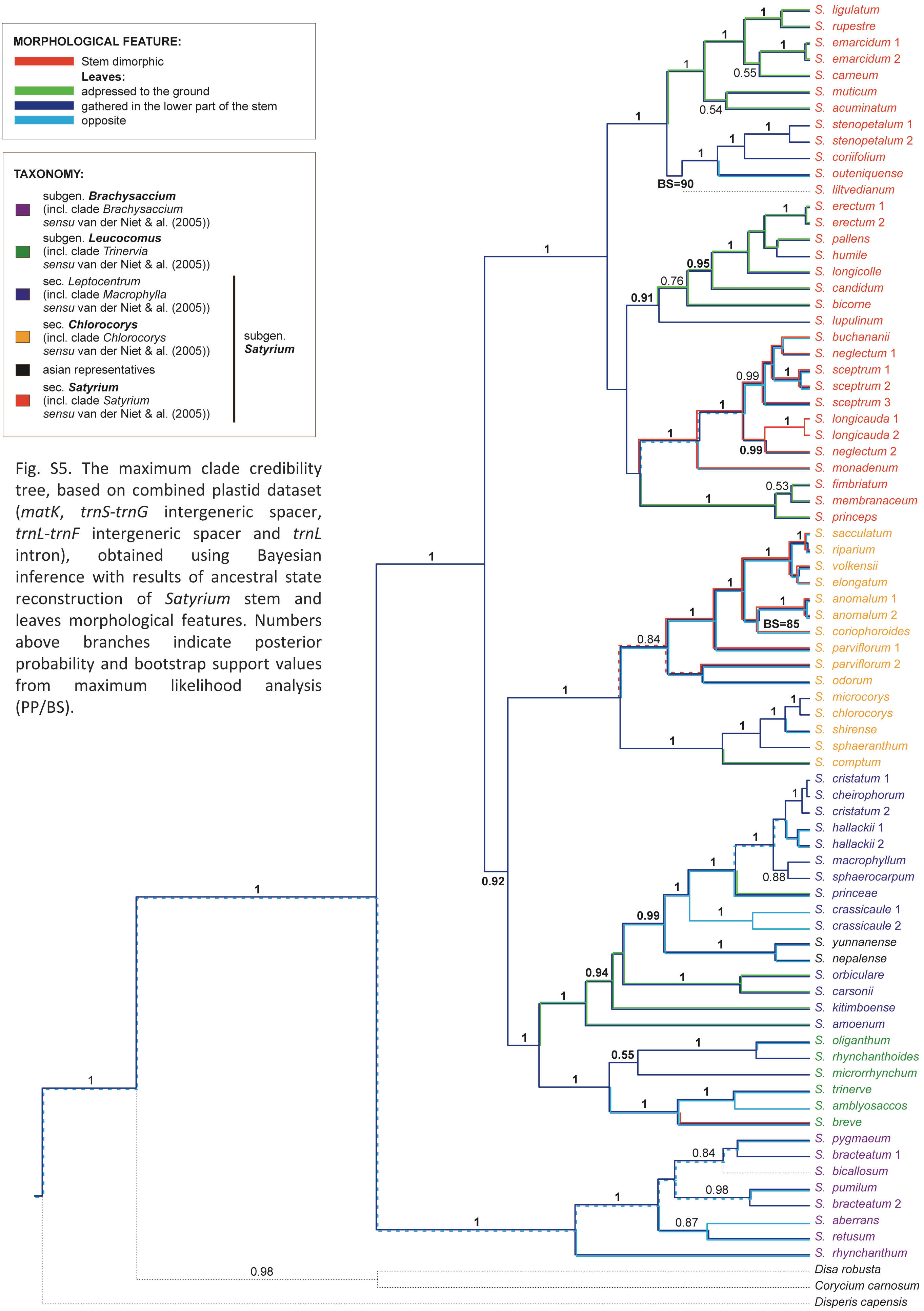

Fig. S5. The maximum clade credibility tree, based on combined plastid dataset (*matK*, *trnS-trnG* intergeneric spacer, *trnL-trnF* intergeneric spacer and *trnL* intron), obtained using Bayesian inference with results of ancestral state reconstruction of *Satyrium* stem and leaves morphological features. Numbers above branches indicate posterior probability and bootstrap support values from maximum likelihood analysis (PP/BS).

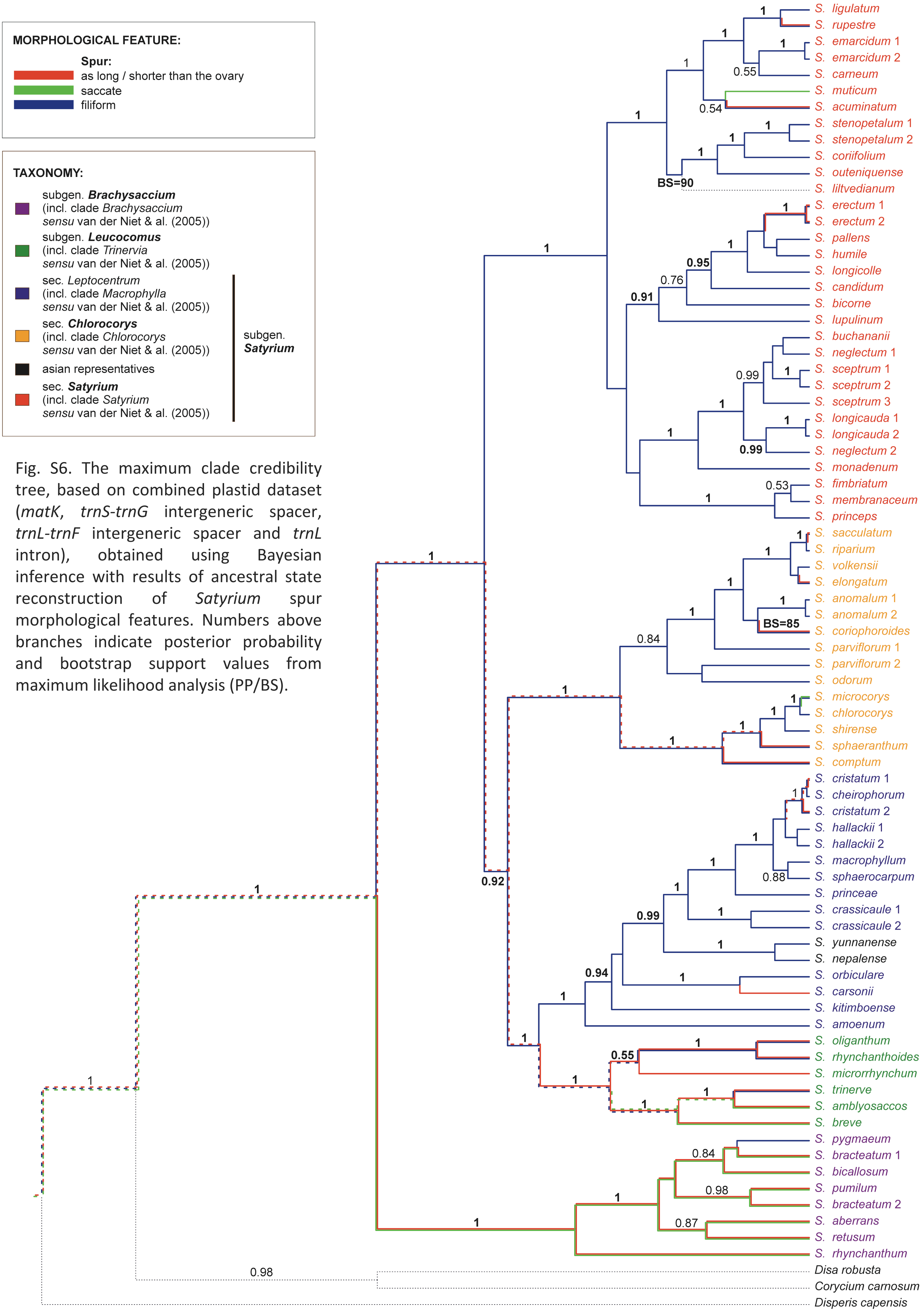

Fig. S6. The maximum clade credibility tree, based on combined plastid dataset (*matK*, *trnS-trnG* intergeneric spacer, *trnL-trnF* intergeneric spacer and *trnL* intron), obtained using Bayesian inference with results of ancestral state reconstruction of *Satyrium* spur morphological features. Numbers above branches indicate posterior probability and bootstrap support values from maximum likelihood analysis (PP/BS).

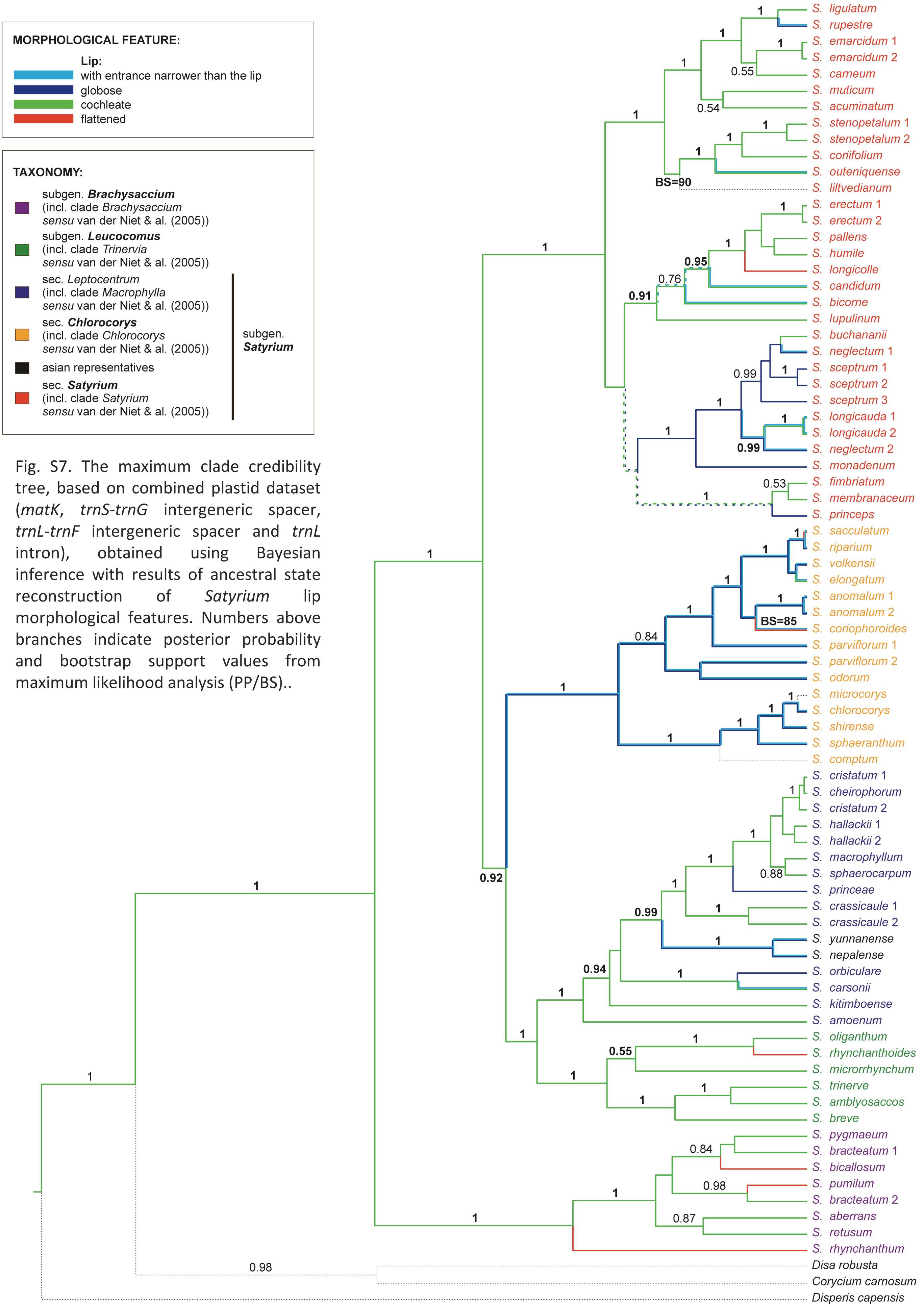

Fig. S7. The maximum clade credibility tree, based on combined plastid dataset (*matK*, *trnS-trnG* intergeneric spacer, *trnL-trnF* intergeneric spacer and *trnL* intron), obtained using Bayesian inference with results of ancestral state reconstruction of *Satyrium* lip morphological features. Numbers above branches indicate posterior probability and bootstrap support values from maximum likelihood analysis (PP/BS)..

Fig. S8. Ancestral range reconstruction of the genus *Satyrium* (nuclear data) estimated using BayArea model.

Letter correspond to the regions: the Cape (A), Natal (B), Zambezian-East Coast (C), Zambezian-South (D), Zambezian-Central (E), Zambesian-West (F), East African Mountains (G), Guinea-Congolian (H), Sudanian (I), Kenyan (J), and Ethiopian Highlands (K), Madagascar (L) and Asia (M).

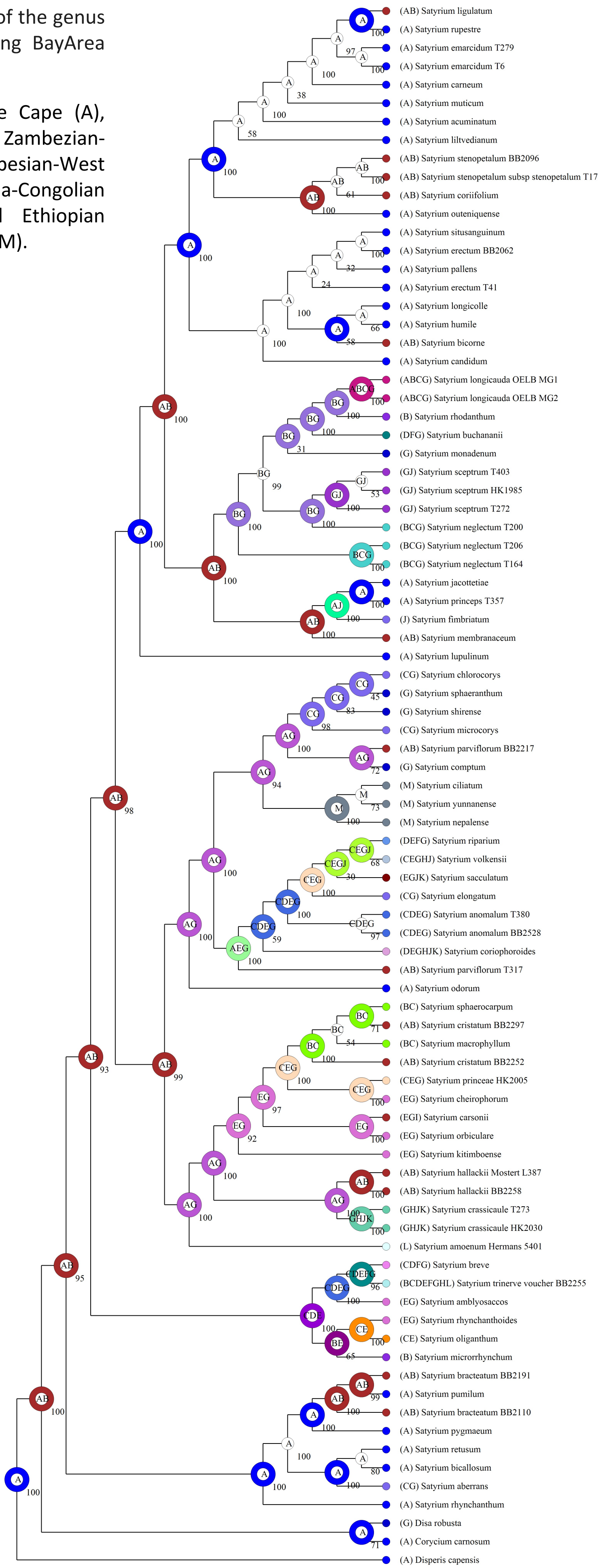

Fig. S9. Ancestral range reconstruction of the genus *Satyrium* (plastid data) estimated using BayArea model.

Letter correspond to the regions: the Cape (A), Natal (B), Zambezian-East Coast (C), Zambezian-South (D), Zambezian-Central (E), Zambesian-West (F), East African Mountains (G), Guinea-Congolian (H), Sudanian (I), Kenyan (J), and Ethiopian Highlands (K), Madagascar (L) and Asia (M).

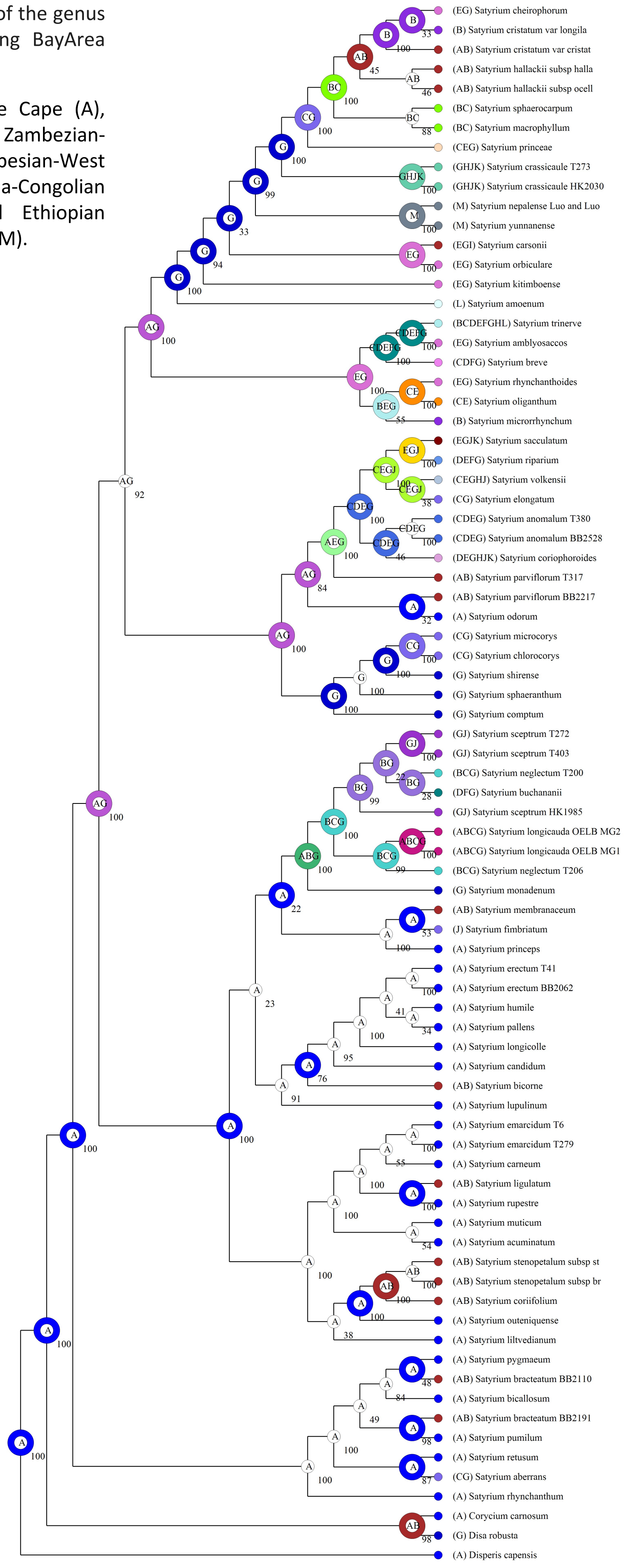

Supplement: Supplementary file 1 [file ijms-27-00453-s001.zip › Supplementary Figures.pdf]
