# Supplementary material for: A Multi-Evidence Approach to the Systematics of the Genus Satyrium Sw. Based on Time-Calibrated Phylogeny, Morphology, and Biogeography
Source: Int J Mol Sci. 2025 Dec 31;27(1):453. doi: 10.3390/ijms27010453 (PMC12787166; doi:10.3390/ijms27010453)
Supplement: Supplementary file 1 [file ijms-27-00453-s001.zip › Supplementary material_S1_BayAreaTest_nuclear.html]

Bayesian Analysis of Biogeography for Satyrium (nuclear)


# Bayesian Analysis of Biogeography for Satyrium (nuclear)

#### Sławomir Nowak

#### 2025-02-17

### MCMC statitics summary

```
## 
## Iterations = 1:6000
## Thinning interval = 1 
## Number of chains = 1 
## Sample size per chain = 6000 
## 
## 1. Empirical mean and standard deviation for each variable,
##    plus standard error of the mean:
## 
##               Mean        SD  Naive SE Time-series SE
## lnL     -1.102e+03 1.224e+02 1.581e+00      9.8986812
## gain     2.424e-02 4.323e-03 5.581e-05      0.0003299
## loss     1.543e-01 2.207e-02 2.849e-04      0.0016225
## gain-p   2.428e-02 4.276e-03 5.520e-05      0.0003332
## loss-p   1.544e-01 2.150e-02 2.776e-04      0.0015746
## distP    2.156e+00 3.769e-01 4.865e-03      0.0229902
## numGain  1.412e+02 2.188e+01 2.824e-01      1.8942679
## numLoss  1.738e+02 2.647e+01 3.417e-01      2.1733166
## 
## 2. Quantiles for each variable:
## 
##               2.5%        25%        50%        75%      97.5%
## lnL     -1.362e+03 -1.182e+03 -1.093e+03 -1.015e+03 -888.47260
## gain     1.679e-02  2.116e-02  2.388e-02  2.696e-02    0.03349
## loss     1.147e-01  1.384e-01  1.533e-01  1.686e-01    0.20090
## gain-p   1.699e-02  2.121e-02  2.397e-02  2.698e-02    0.03348
## loss-p   1.159e-01  1.390e-01  1.529e-01  1.686e-01    0.19931
## distP    1.428e+00  1.899e+00  2.148e+00  2.410e+00    2.90601
## numGain  1.040e+02  1.260e+02  1.390e+02  1.550e+02  189.00000
## numLoss  1.290e+02  1.540e+02  1.720e+02  1.910e+02  231.02500
```

### Traceplot and densplot of MCMC

### Samples autocorrelation

### Geweke diagnostics

```
## 
## Fraction in 1st window = 0.1
## Fraction in 2nd window = 0.5 
## 
##     lnL    gain    loss  gain-p  loss-p   distP numGain numLoss 
##  0.7360 -1.1209 -1.7347 -1.0460 -1.4290  0.4306 -1.2131 -0.2797
```

### Effective samples size

```
##      lnL     gain     loss   gain-p   loss-p    distP  numGain  numLoss 
## 152.9701 171.7466 184.9526 164.6569 186.5155 268.7276 133.3849 148.3096
```

### Heidelberger and Welch diagnostics

```
##                                       
##         Stationarity start     p-value
##         test         iteration        
## lnL     passed       1         0.385  
## gain    passed       1         0.280  
## loss    passed       1         0.212  
## gain-p  passed       1         0.243  
## loss-p  passed       1         0.239  
## distP   passed       1         0.640  
## numGain passed       1         0.248  
## numLoss passed       1         0.474  
##                                      
##         Halfwidth Mean      Halfwidth
##         test                         
## lnL     passed    -1.10e+03 1.94e+01 
## gain    passed     2.42e-02 6.47e-04 
## loss    passed     1.54e-01 3.18e-03 
## gain-p  passed     2.43e-02 6.53e-04 
## loss-p  passed     1.54e-01 3.09e-03 
## distP   passed     2.16e+00 4.51e-02 
## numGain passed     1.41e+02 3.71e+00 
## numLoss passed     1.74e+02 4.26e+00
```

### Raftery and Lewis diagnostics

```
## 
## Quantile (q) = 0.025
## Accuracy (r) = +/- 0.005
## Probability (s) = 0.95 
##                                                
##          Burn-in  Total Lower bound  Dependence
##          (M)      (N)   (Nmin)       factor (I)
##  lnL     22       24026 3746         6.41      
##  gain    12       12660 3746         3.38      
##  loss    12       12922 3746         3.45      
##  gain-p  6        6940  3746         1.85      
##  loss-p  10       10544 3746         2.81      
##  distP   12       14184 3746         3.79      
##  numGain 24       31060 3746         8.29      
##  numLoss 16       19614 3746         5.24
```
