# Supplementary material for: A Multi-Evidence Approach to the Systematics of the Genus Satyrium Sw. Based on Time-Calibrated Phylogeny, Morphology, and Biogeography
Source: Int J Mol Sci. 2025 Dec 31;27(1):453. doi: 10.3390/ijms27010453 (PMC12787166; doi:10.3390/ijms27010453)
Supplement: Supplementary file 1 [file ijms-27-00453-s001.zip › Supplementary material_S2_BayAreaTest_plastids.html]

Bayesian Analysis of Biogeography for Satyrium (plastids)


# Bayesian Analysis of Biogeography for Satyrium (plastids)

#### Sławomir Nowak

#### 2025-02-17

### MCMC statitics summary

```
## 
## Iterations = 1:6000
## Thinning interval = 1 
## Number of chains = 1 
## Sample size per chain = 6000 
## 
## 1. Empirical mean and standard deviation for each variable,
##    plus standard error of the mean:
## 
##               Mean        SD  Naive SE Time-series SE
## lnL     -953.22771 1.085e+02 1.401e+00      9.1916088
## gain       0.02448 4.429e-03 5.718e-05      0.0003440
## loss       0.15757 2.466e-02 3.183e-04      0.0016044
## gain-p     0.02447 4.289e-03 5.537e-05      0.0003185
## loss-p     0.15756 2.447e-02 3.159e-04      0.0017985
## distP      2.15806 3.702e-01 4.780e-03      0.0191085
## numGain  126.22900 1.917e+01 2.475e-01      1.6812495
## numLoss  146.08817 2.488e+01 3.212e-01      2.0196081
## 
## 2. Quantiles for each variable:
## 
##               2.5%        25%        50%        75%      97.5%
## lnL     -1.177e+03 -1.024e+03 -947.38250 -878.41800 -756.56855
## gain     1.678e-02  2.127e-02    0.02418    0.02740    0.03366
## loss     1.116e-01  1.405e-01    0.15647    0.17367    0.20798
## gain-p   1.699e-02  2.141e-02    0.02421    0.02718    0.03369
## loss-p   1.131e-01  1.401e-01    0.15639    0.17374    0.20910
## distP    1.459e+00  1.903e+00    2.14708    2.40514    2.89396
## numGain  9.300e+01  1.130e+02  125.00000  139.00000  166.00000
## numLoss  1.010e+02  1.290e+02  145.00000  161.00000  199.00000
```

### Traceplot and densplot of MCMC

### Samples autocorrelation

### Geweke diagnostics

```
## 
## Fraction in 1st window = 0.1
## Fraction in 2nd window = 0.5 
## 
##     lnL    gain    loss  gain-p  loss-p   distP numGain numLoss 
##  0.5647 -0.4117 -1.3298 -0.4796 -0.9919  0.7564 -0.3467 -0.5927
```

### Effective samples size

```
##      lnL     gain     loss   gain-p   loss-p    distP  numGain  numLoss 
## 139.4670 165.7082 236.1768 181.3914 185.0664 375.4143 130.0029 151.7617
```

### Heidelberger and Welch diagnostics

```
##                                       
##         Stationarity start     p-value
##         test         iteration        
## lnL     passed          1      0.0929 
## gain    passed          1      0.1559 
## loss    passed       1201      0.0521 
## gain-p  passed          1      0.1394 
## loss-p  passed          1      0.1766 
## distP   passed          1      0.5117 
## numGain passed          1      0.1002 
## numLoss passed          1      0.1281 
##                                      
##         Halfwidth Mean      Halfwidth
##         test                         
## lnL     passed    -953.2277 1.80e+01 
## gain    passed       0.0245 6.74e-04 
## loss    passed       0.1584 3.53e-03 
## gain-p  passed       0.0245 6.24e-04 
## loss-p  passed       0.1576 3.53e-03 
## distP   passed       2.1581 3.75e-02 
## numGain passed     126.2290 3.30e+00 
## numLoss passed     146.0882 3.96e+00
```

### Raftery and Lewis diagnostics

```
## 
## Quantile (q) = 0.025
## Accuracy (r) = +/- 0.005
## Probability (s) = 0.95 
##                                                
##          Burn-in  Total Lower bound  Dependence
##          (M)      (N)   (Nmin)       factor (I)
##  lnL     22       22220 3746         5.93      
##  gain    12       12764 3746         3.41      
##  loss    18       19569 3746         5.22      
##  gain-p  18       18606 3746         4.97      
##  loss-p  20       21356 3746         5.70      
##  distP   9        11793 3746         3.15      
##  numGain 18       20202 3746         5.39      
##  numLoss 28       32656 3746         8.72
```
